# Supplementary figures and images for: Pre-CRRT furosemide and mortality in sepsis-associated AKI: A retrospective cohort study
Source: PLoS One. 2026 Apr 20;21(4):e0347094. doi: 10.1371/journal.pone.0347094 (PMC13095019; doi:10.1371/journal.pone.0347094)

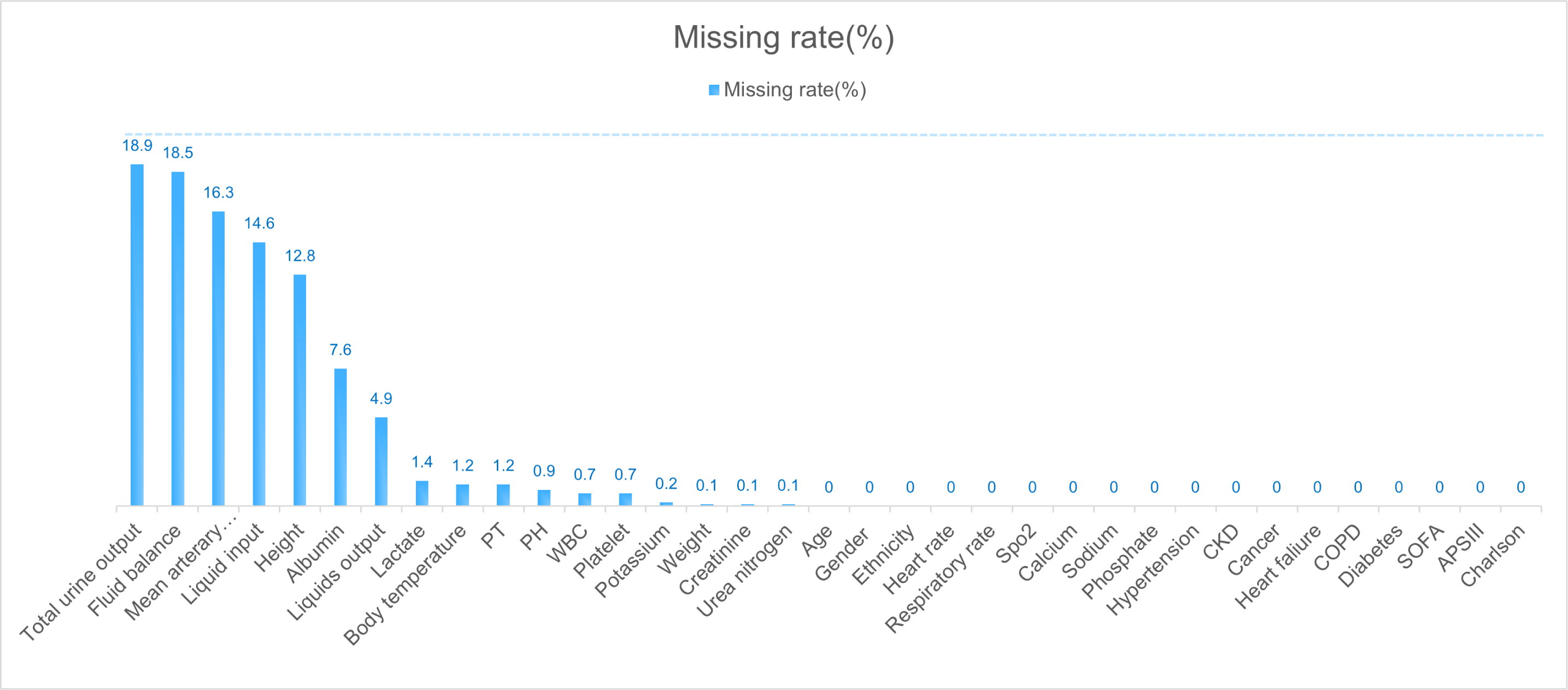

Supplement: S1 Fig — Abbreviations: MAP: Mean arterial pressure; WBC: White blood cell; PT: Prothrombin Time; CKD: Chronic kidney disease; COPD: Chronic Obstructive Pulmonary Disease; SOFA: Sequential Organ Failure Assessment score; APACHEII: Acute Physiology and Chronic Health Evaluation II score; CCI: Charlson Comorbidity Index. (TIF) [file pone.0347094.s001.tif]

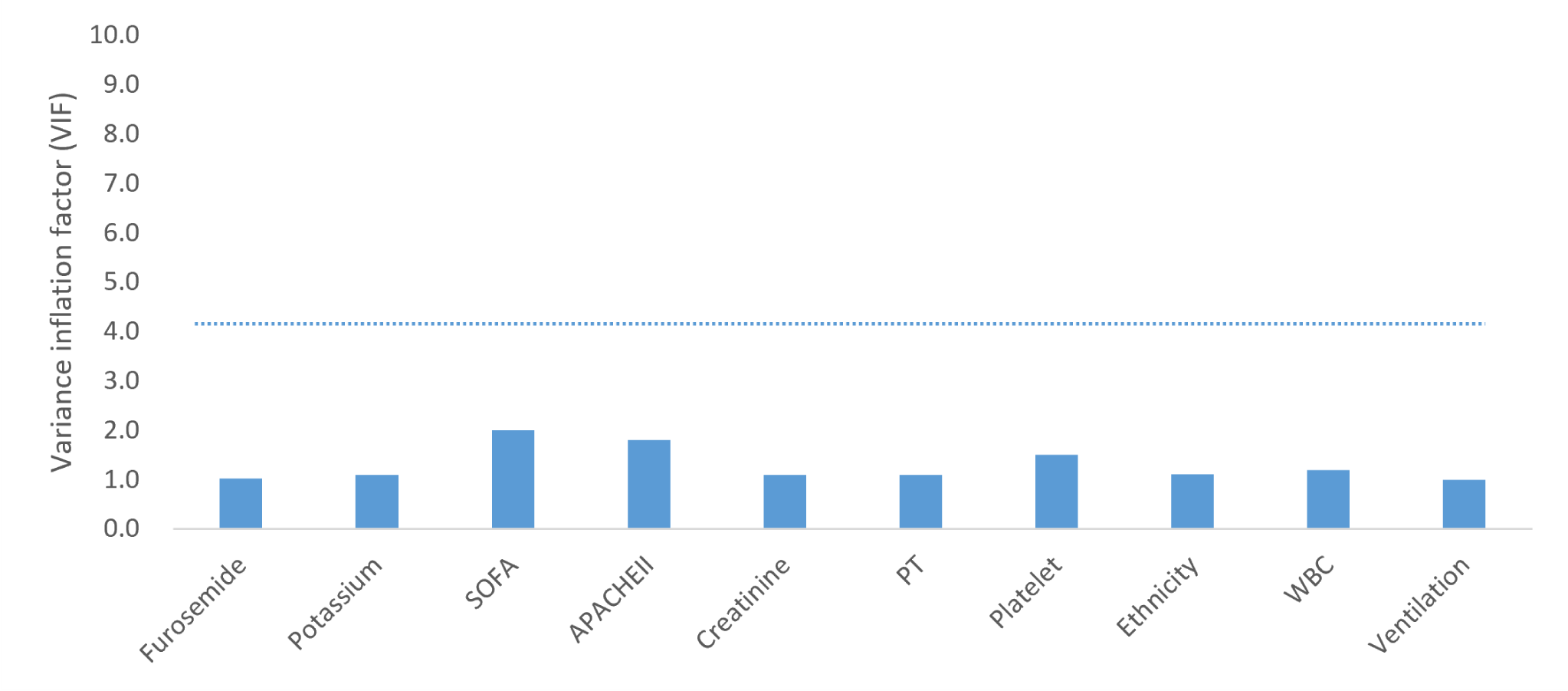

Supplement: S2 Fig — Abbreviations: WBC: White blood cell; PT: Prothrombin Time; SOFA: Sequential Organ Failure Assessment score; APACHEII: Acute Physiology and Chronic Health Evaluation II score; A variance inflation factor of <5 for each variable suggested the absence of multicollinearity. (TIF) [file pone.0347094.s002.tif]

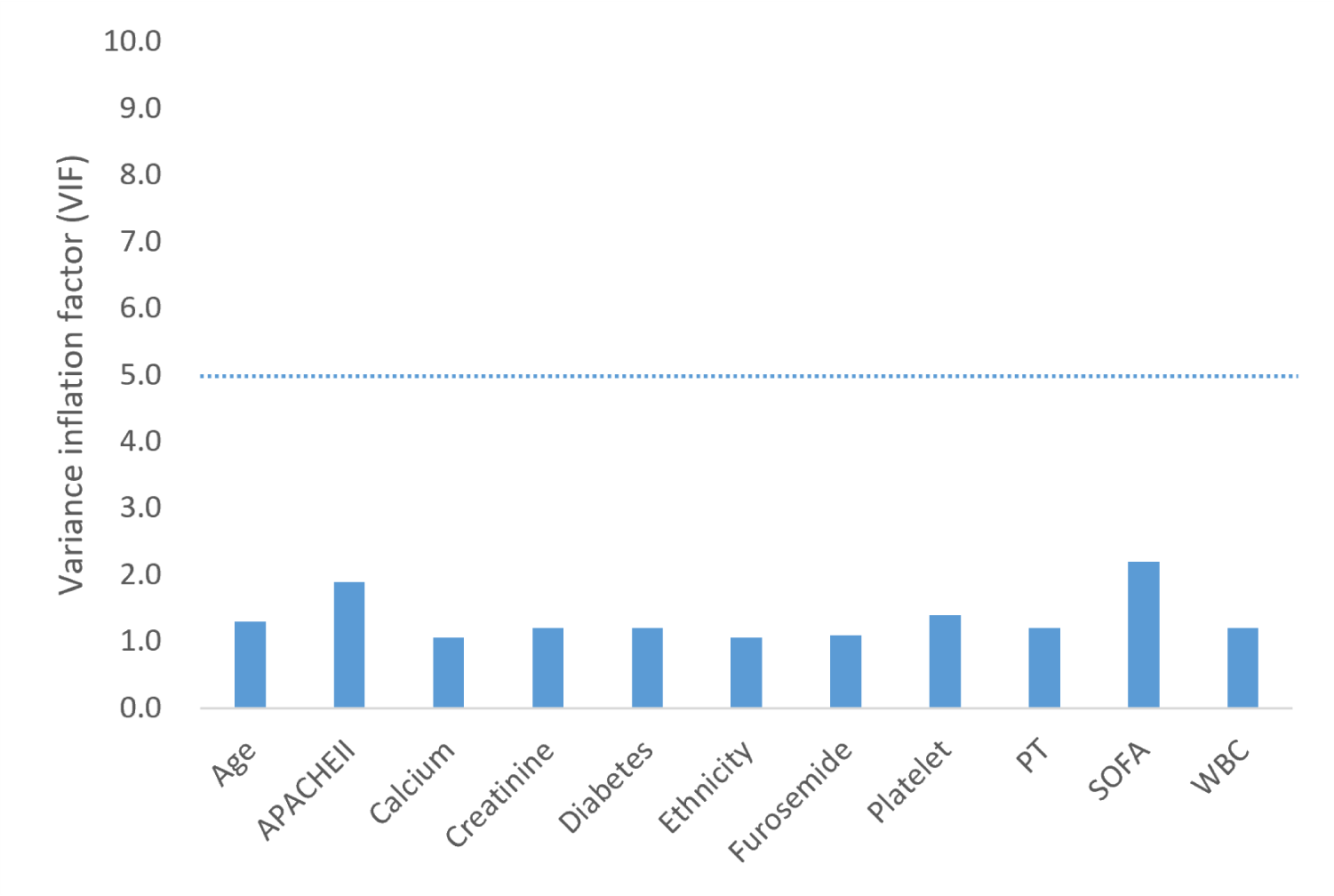

Supplement: S3 Fig — Abbreviations: WBC: White blood cell; PT: Prothrombin Time; SOFA: Sequential Organ Failure Assessment score; APACHEII: Acute Physiology and Chronic Health Evaluation II score; A variance inflation factor of <5 for each variable suggested the absence of multicollinearity. (TIF) [file pone.0347094.s003.tif]

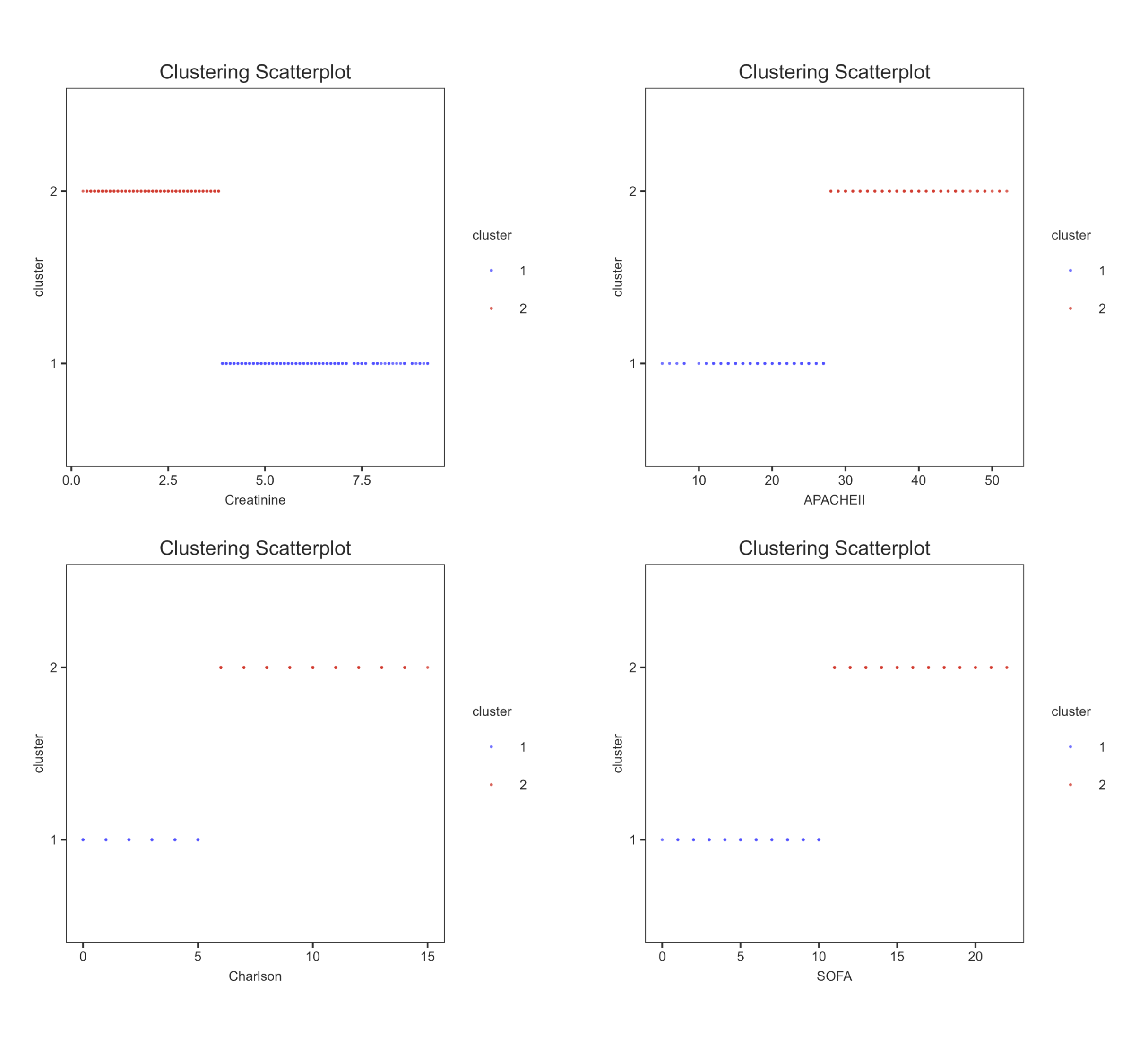

Supplement: S4 Fig — Abbreviations: SOFA: Sequential Organ Failure Assessment score; APACHEII: Acute Physiology and Chronic Health Evaluation II score; Charlson score: Charlson Comorbidity Index. (TIF) [file pone.0347094.s004.tif]

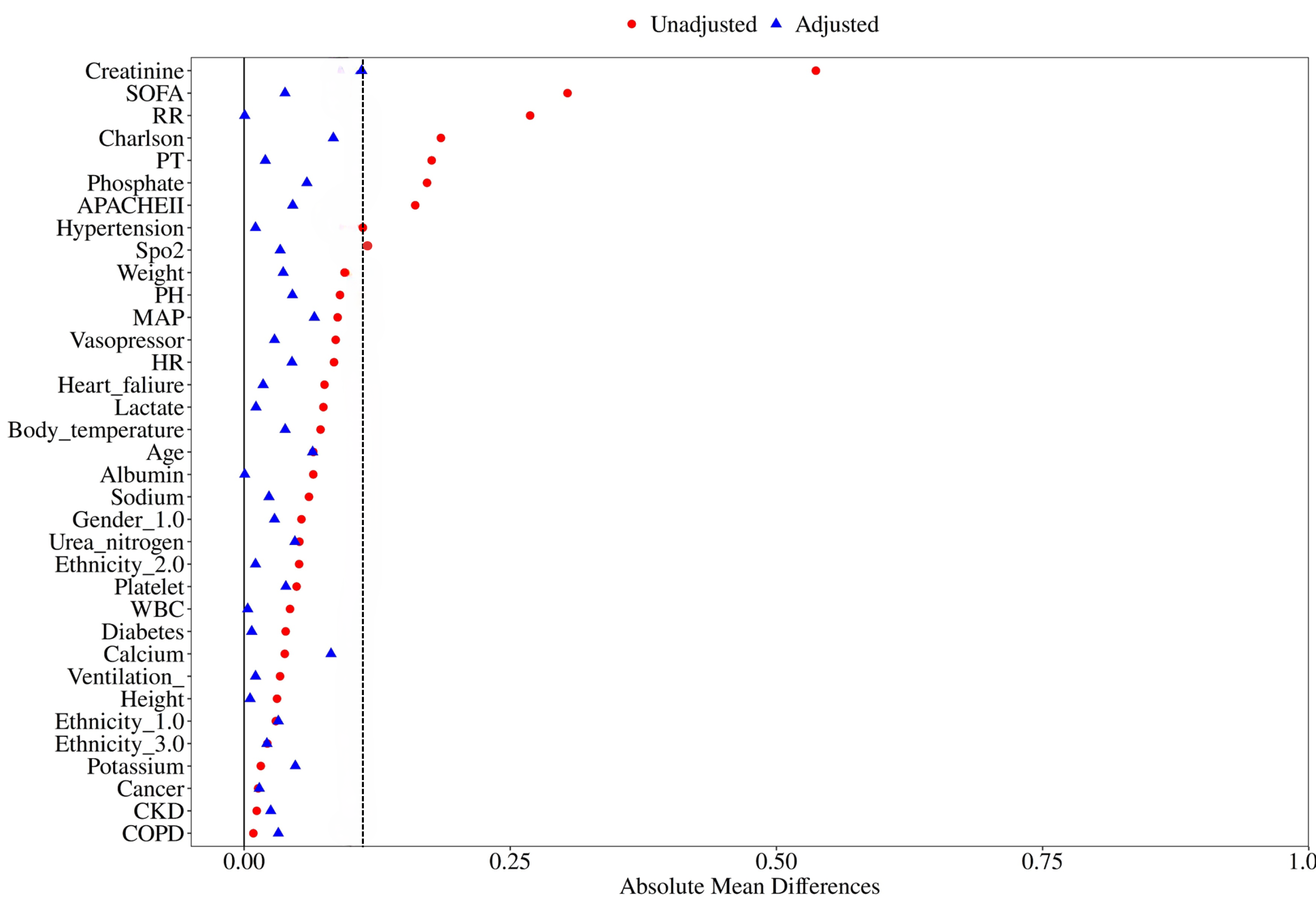

Supplement: S5 Fig — Abbreviations: MAP: Mean arterial pressure; WBC: White blood cell; PT: Prothrombin Time; CKD: Chronic kidney disease; COPD: Chronic Obstructive Pulmonary Disease; SOFA: Sequential Organ Failure Assessment score; APACHEII: Acute Physiology and Chronic Health Evaluation II score; Ethnicity_1.0: White; Ethnicity_2.0: Black; Ethnicity_3.0: Other. (TIF) [file pone.0347094.s005.tif]

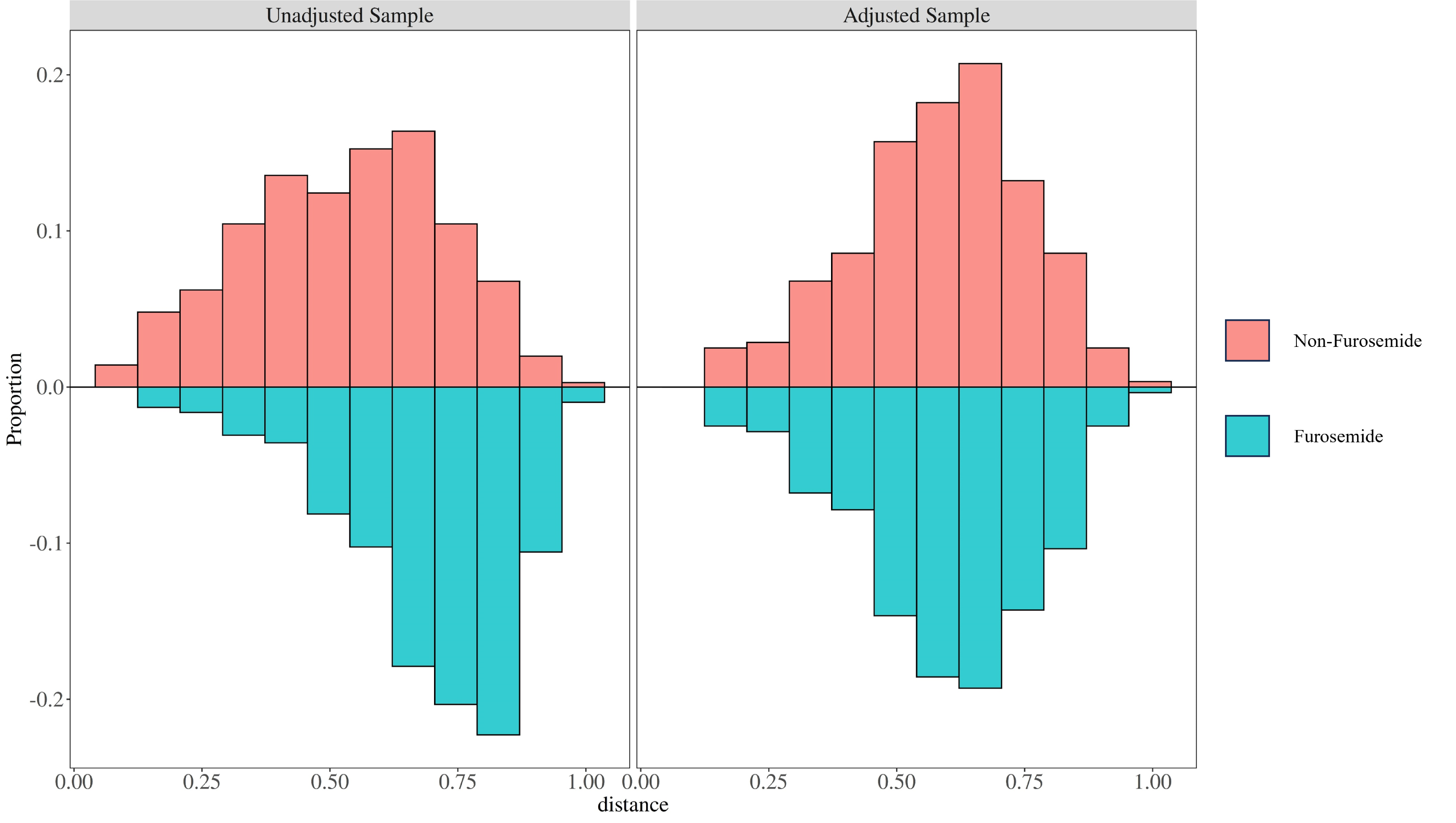

Supplement: S6 Fig — (TIF) [file pone.0347094.s006.tif]

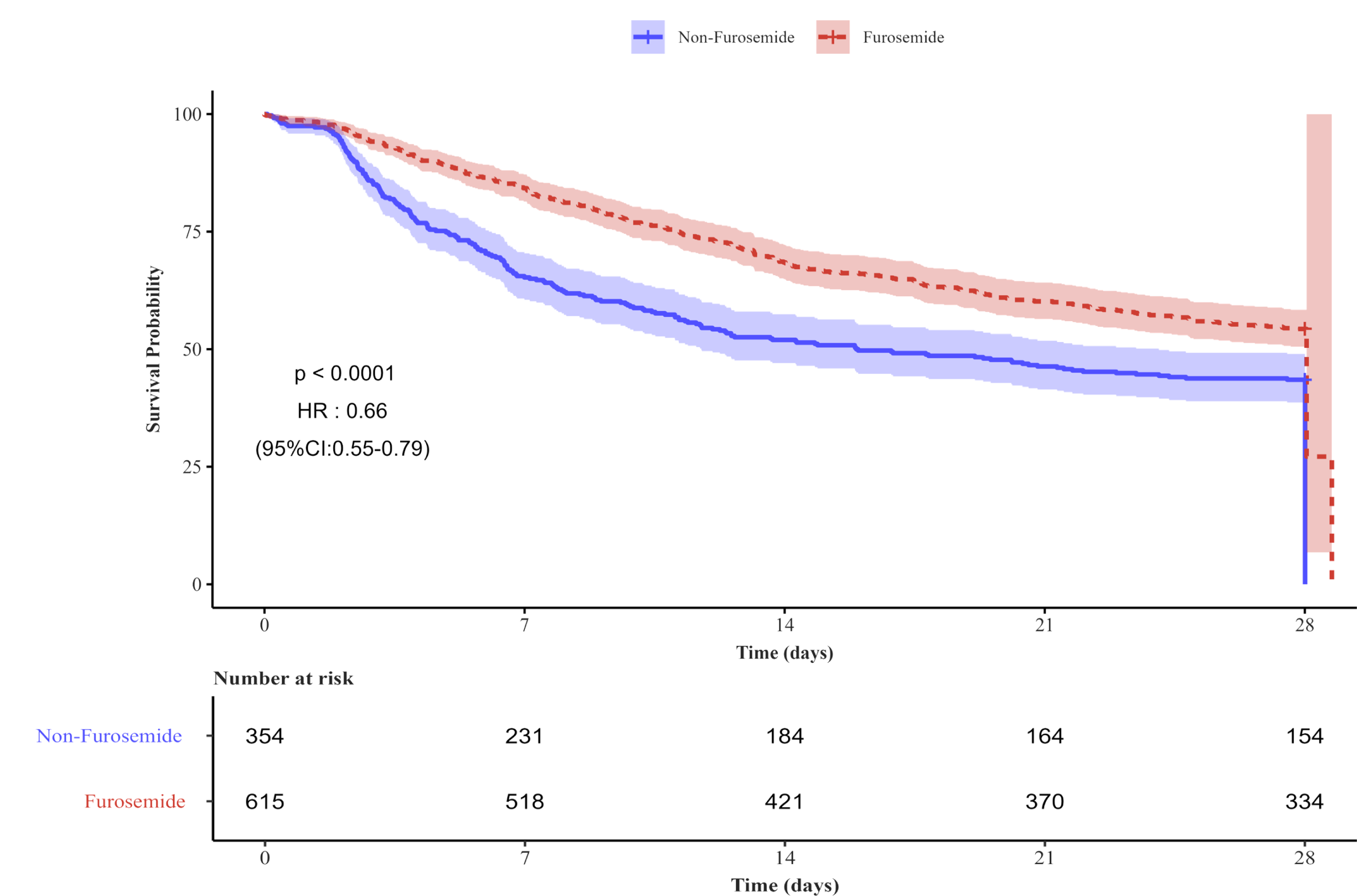

Supplement: S7 Fig — (TIF) [file pone.0347094.s007.tif]

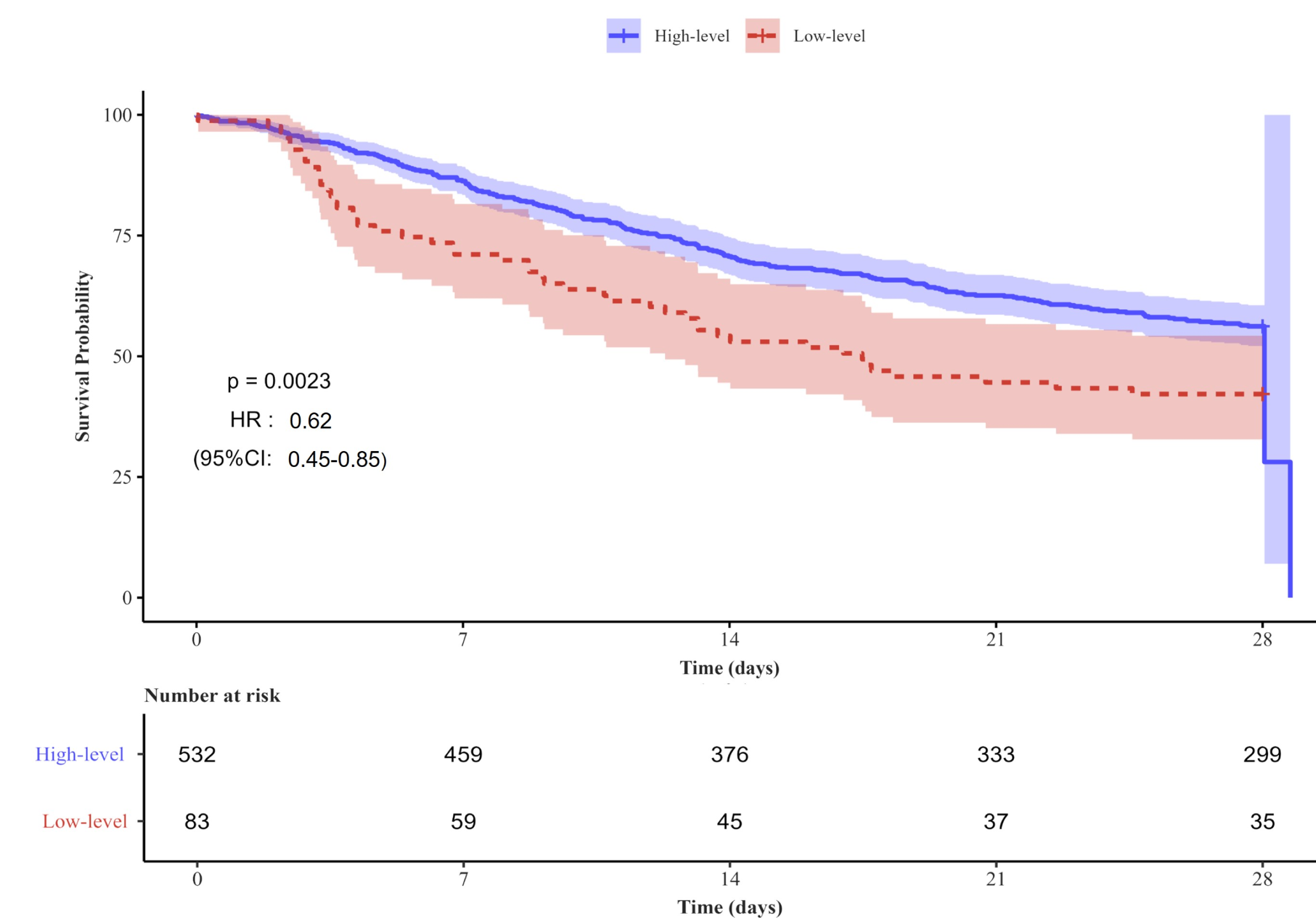

Supplement: S8 Fig — (TIF) [file pone.0347094.s008.tif]

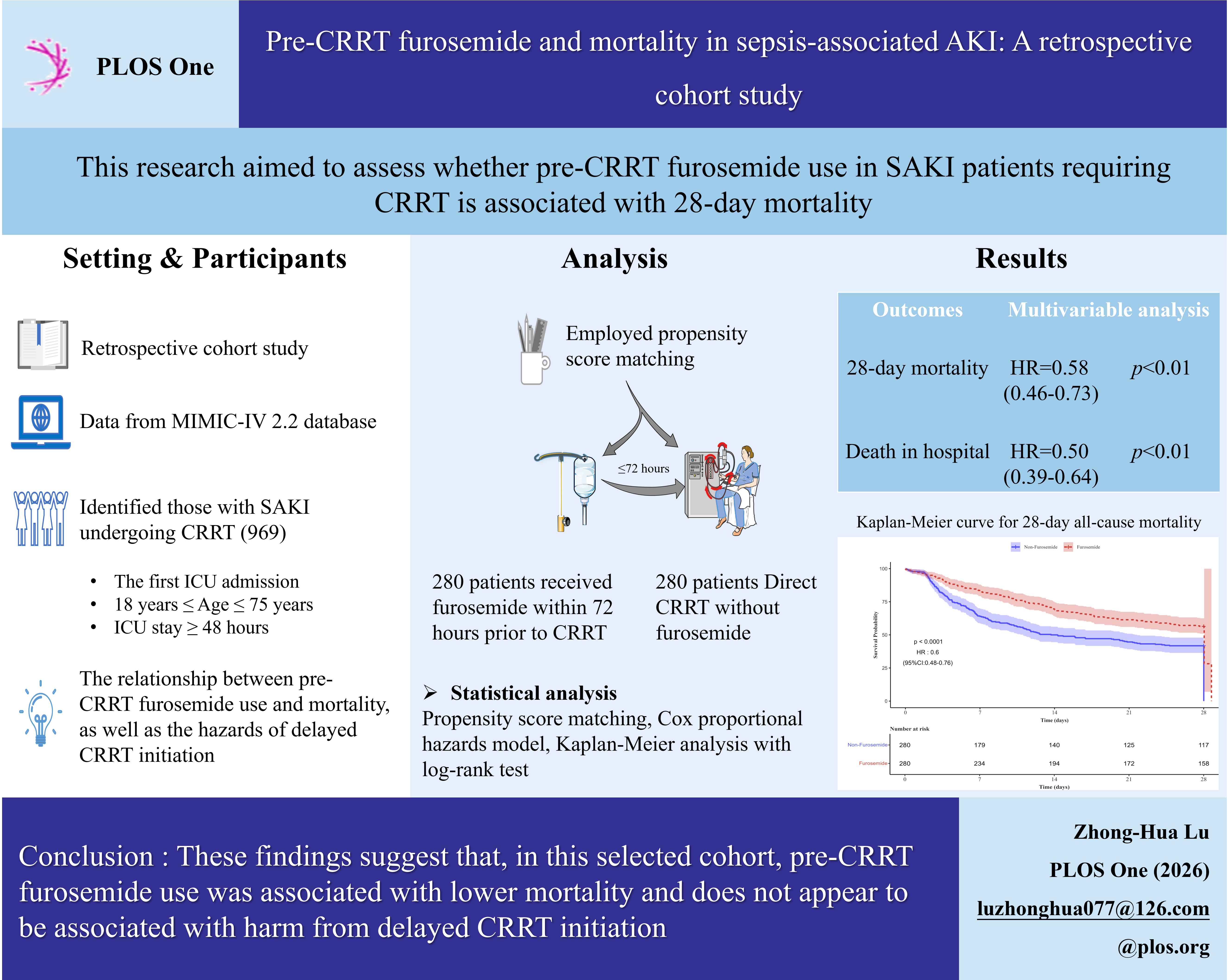

Supplement: S9 Fig — This retrospective cohort study utilized the MIMIC-IV 2.2 database to enroll 969 patients with SAKI who were first admitted to the ICU, aged between 18 and 75 years, had a hospital stay of 48 hours or more, and required CRRT. Through PSM, 280 patients who received furosemide within 72 hours prior to CRRT were paired with 280 patients who initiated CRRT directly without the use of furosemide. The primary outcome measured was 28-day mortality (HR = 0.58, 95% CI 0.46–0.73), while the secondary outcome was in-hospital mortality (HR = 0.50, 95% CI 0.39–0.64). The Kaplan-Meier curve illustrated a lower 28-day all-cause mortality rate in the furosemide group. In conclusion, within the selected cohort, the use of furosemide prior to CRRT was associated with lower mortality, with no observed harm from a delay in CRRT initiation. Abbreviations: AKI: Acute kidney injury; SAKI: Sepsis-associated acute kidney injury; CRRT: Continuous renal replacement therapy; ICU: Intensive care unit. (TIF) [file pone.0347094.s009.tif]
