# Supplementary material for: Pre-CRRT furosemide and mortality in sepsis-associated AKI: A retrospective cohort study
Source: PLoS One. 2026 Apr 20;21(4):e0347094. doi: 10.1371/journal.pone.0347094 (PMC13095019; doi:10.1371/journal.pone.0347094)
Supplement: S1 Table — Abbreviations: MAP: Mean arterial pressure; WBC: White blood cell; PT: Prothrombin Time; CKD: Chronic kidney disease; COPD: Chronic Obstructive Pulmonary Disease; SOFA: Sequential Organ Failure Assessment score; APACHEII: Acute Physiology and Chronic Health Evaluation II score; CCI: Charlson Comorbidity Index. (DOCX) [file pone.0347094.s010.docx]

**Table S1. Percentage of missing data of each variable.**

| Variables | Missing rate (%) |
| --- | --- |
| Age | 0.0 |
| Gender | 0.0 |
| Ethnicity | 0.0 |
| Weight | 0.1 |
| Height | 12.8 |
| Heart rate | 0.0 |
| Respiratory rate | 0.0 |
| Mean arterary pressure | 16.3 |
| Body temperature | 1.2 |
| Spo2 | 0.0 |
| Creatinine | 0.1 |
| Urea nitrogen | 0.1 |
| Lactate | 1.4 |
| PH | 0.9 |
| Calcium | 0.0 |
| Potassium | 0.2 |
| Sodium | 0.0 |
| Phosphate | 0.0 |
| PT | 1.2 |
| WBC | 0.7 |
| Platelet | 0.7 |
| Albumin | 7.6 |
| Total urine output | 18.9 |
| Liquid input | 14.6 |
| Liquids output | 4.9 |
| Fluid balance | 18.5 |
| Hypertension | 0.0 |
| CKD | 0.0 |
| Cancer | 0.0 |
| Heart faliure | 0.0 |
| COPD | 0.0 |
| Diabetes | 0.0 |
| SOFA | 0.0 |
| APACHEII | 0.0 |
| CCI | 0.0 |
| Furosemide | 0.0 |
| Ventilation | 0.0 |
| Vasopressors | 0.0 |

*Abbreviations: MAP: Mean arterial pressure; WBC: White blood cell; PT: Prothrombin Time; CKD: Chronic kidney disease; COPD: Chronic Obstructive Pulmonary Disease; SOFA: Sequential Organ Failure Assessment score; APACHEII: Acute Physiology and Chronic Health Evaluation II score; CCI: Charlson Comorbidity Index.*
