# Supplementary material for: Pre-CRRT furosemide and mortality in sepsis-associated AKI: A retrospective cohort study
Source: PLoS One. 2026 Apr 20;21(4):e0347094. doi: 10.1371/journal.pone.0347094 (PMC13095019; doi:10.1371/journal.pone.0347094)
Supplement: S2 Table — Abbreviations: WBC: White blood cell; PT: Prothrombin Time; SOFA: Sequential Organ Failure Assessment score; APACHEII: Acute Physiology and Chronic Health Evaluation II score; A variance inflation factor of <5 for each variable suggested the absence of multicollinearity. (DOCX) [file pone.0347094.s011.docx]

**Table S2. Variance inflation factor of each variable in the matched cohort.**

| Variables | Variance inflation factor (VIF) |
| --- | --- |
| Furosemide | 1.0 |
| Potassium | 1.1 |
| SOFA | 2.0 |
| APACHEII | 1.8 |
| Creatinine | 1.1 |
| PT | 1.1 |
| Platelet | 1.5 |
| Ethnicity | 1.1 |
| WBC | 1.2 |
| Ventilation | 1.0 |

*Abbreviations: WBC: White blood cell; PT: Prothrombin Time; SOFA: Sequential Organ Failure Assessment score; APACHEII: Acute Physiology and Chronic Health Evaluation II score; A variance inflation factor of <5 for each variable suggested the absence of multicollinearity.*
