# Supplementary material for: Pre-CRRT furosemide and mortality in sepsis-associated AKI: A retrospective cohort study
Source: PLoS One. 2026 Apr 20;21(4):e0347094. doi: 10.1371/journal.pone.0347094 (PMC13095019; doi:10.1371/journal.pone.0347094)
Supplement: S4 Table — Abbreviations: SOFA: Sequential Organ Failure Assessment score; APACHEII: Acute Physiology and Chronic Health Evaluation II score; Charlson score: Charlson Comorbidity Index. (DOCX) [file pone.0347094.s013.docx]

**Table S4. K-Means clustering analysis.**

| Variables | Cluster Level | Cluster Mean | Cluster Size |
| --- | --- | --- | --- |
| SOFA | Low | 7.36 | 438 |
|  | High | 13.94 | 531 |
| Charlson score | Low | 3.29 | 518 |
|  | High | 7.97 | 451 |
| APACHEII | Low | 21.64 | 491 |
|  | High | 33.54 | 478 |
| Creatinine | Low | 2.01 | 683 |
|  | High | 5.66 | 286 |

*Abbreviations: SOFA: Sequential Organ Failure Assessment score; APACHEII: Acute Physiology and Chronic Health Evaluation II score; Charlson score: Charlson Comorbidity Index.*
