# Supplementary material for: Pre-CRRT furosemide and mortality in sepsis-associated AKI: A retrospective cohort study
Source: PLoS One. 2026 Apr 20;21(4):e0347094. doi: 10.1371/journal.pone.0347094 (PMC13095019; doi:10.1371/journal.pone.0347094)
Supplement: S5 Table — Abbreviations: MAP: Mean arterial pressure; WBC: White blood cell; PT: Prothrombin Time; CKD: Chronic kidney disease; COPD: Chronic Obstructive Pulmonary Disease; SOFA: Sequential Organ Failure Assessment score; APACHEII: Acute Physiology and Chronic Health Evaluation II score; CCI: Charlson Comorbidity Index. a Total urine output in the 72 hours prior to CRRT. b The most recent fluid intake and output record and fluid balance record before CRRT. c Requirement of vasoactive drugs or mechanical ventilation on the first day of ICU admission. (DOCX) [file pone.0347094.s014.docx]

**Table S5. Baseline characteristics before propensity score matching.**

|  | | Group | |  | |
| --- | --- | --- | --- | --- | --- |
| Variable | Overall | Non-Furosemide | Furosemide | *p*-value | SMD |
| N | 969 | 354 | 615 |  |  |
| Age (years), median (IQR) | 60 (50-68) | 59 (50-67) | 61 (49-68) | 0.33 | 0.07 |
| Male (%), median (IQR) | 597 (61.61) | 206 (58.19) | 391 (63.58) | 0.11 | 0.11 |
| Ethnicity (%), median (IQR) |  |  |  | 0.07 | 0.15 |
| White | 522 (53.87) | 184 (51.98) | 338 (54.96) |  |  |
| Black | 127 (13.11) | 58 (16.38) | 69 (11.22) |  |  |
| Other | 320 (33.02) | 112 (31.64) | 208 (33.82) |  |  |
| Height (cm), median (IQR) | 170 (165-178) | 170 (165-178) | 170 (165-178) | 0.64 | 0.03 |
| Weight (kg), median (IQR) | 88 (74.3-102.1) | 87.9 (72.71-100.85) | 88 (75.8-104.18) | 0.15 | 0.10 |
| Heart rate (/bpm), median (IQR) | 95 (81-110) | 94 (80-108) | 95 (82-112) | 0.22 | 0.08 |
| Respiratory rate (/bpm), median (IQR) | 21 (17-25) | 20 (17-24) | 21 (18-26) | <0.01 | 0.27 |
| Mean arterial pressure (mmHg), median (IQR) | 74 (65-85) | 74 (64-85) | 74 (66-85) | 0.20 | 0.09 |
| Body temperature (℃), median (IQR) | 36.78 (36.5-37.06) | 36.78 (36.5-37) | 36.78 (36.5-37.06) | 0.28 | 0.07 |
| Spo2 (%), median (IQR) | 97 (94-100) | 97 (95-100) | 97 (94-100) | 0.12 | 0.10 |
| WBC (×10^9^/L), median (IQR) | 12.5 (7.9-17.6) | 12.5 (7.4-18.4) | 12.5 (8.1-17.2) | 0.53 | 0.04 |
| Albumin (g/dL), median (IQR) | 2.8 (2.3-3.2) | 2.7 (2.3-3.2) | 2.8 (2.4-3.2) | 0.35 | 0.06 |
| Platelet (×10^9^/L), median (IQR) | 140 (88-214) | 134.5 (80-217) | 140 (93.5-211.5) | 0.47 | 0.05 |
| PT (seconds), median (IQR) | 16.2 (13.8-20.7) | 16.3 (14.3-21.32) | 16.2 (13.6-20.25) | 0.01 | 0.17 |
| Lactate (mmol/L), median (IQR) | 2.3 (1.5-3.7) | 2.3 (1.6-3.7) | 2.3 (1.5-3.75) | 0.27 | 0.07 |
| PH, median (IQR) | 7.31 (7.21-7.38) | 7.31 (7.2-7.38) | 7.31 (7.23-7.38) | 0.20 | 0.08 |
| Creatinine (mg/dL), median (IQR) | 2.6 (1.5-4.2) | 3.3 (2.1-5.18) | 2.2 (1.3-3.7) | <0.01 | 0.51 |
| Urea nitrogen (mg/dL), median (IQR) | 37 (23-59) | 38 (25-57) | 36 (22-59.5) | 0.42 | 0.05 |
| Calcium (mg/dL), median (IQR) | 8.1 (7.5-8.7) | 8.15 (7.4-8.9) | 8.1 (7.5-8.7) | 0.58 | 0.04 |
| Potassium (mEq/L), median (IQR) | 4.4 (3.9-5) | 4.4 (3.9-5.07) | 4.4 (3.9-5) | 0.81 | 0.02 |
| Sodium(mEq/L), median (IQR) | 137 (133-140) | 136 (133-140) | 137 (133-141) | 0.35 | 0.06 |
| Phosphate (mg/dL), median (IQR) | 5 (3.7-6.6) | 5.1 (3.9-6.97) | 4.9 (3.6-6.5) | 0.01 | 0.17 |
| Total urine output^a^ (ml), median (IQR) | 445 (138-1052) | 215 (50.25-628) | 495 (259.5-1315.5) | <0.01 | 0.52 |
| Liquid input^b^ (ml), median (IQR) | 4650.92 (2480-8224.71) | 4833.93 (2802.07-8834.28) | 4541.66 (2262-7976.26) | 0.04 | 0.14 |
| Liquid output^b^ (ml), median (IQR) | 885 (375-1753) | 650 (240-1217.5) | 1009 (500-1956) | <0.01 | 0.33 |
| Fluid balance^b^ (ml), median (IQR) | 3318.01 (1160.73-6547.47) | 3955.87 (2007.98-7188.91) | 3134.92 (868.16-6167.68) | <0.01 | 0.23 |
| Total dose of furosemide | / | / | 340 (160-740) | / | / |
| Hypertension, n (%) | 252 (26.01) | 67 (18.93) | 185 (30.08) | <0.01 | 0.26 |
| CKD, n (%) | 229 (23.63) | 81 (22.88) | 148 (24.07) | 0.73 | 0.03 |
| Cancer, n (%) | 74 (7.64) | 30 (8.47) | 44 (7.15) | 0.54 | 0.05 |
| Heart failure, n (%) | 301 (31.06) | 93 (26.27) | 208 (33.82) | 0.02 | 0.17 |
| COPD, n (%) | 134 (13.83) | 47 (13.28) | 87 (14.15) | 0.78 | 0.03 |
| Diabetes, n (%) | 347 (35.81) | 118 (33.33) | 229 (37.24) | 0.25 | 0.08 |
| SOFA, median (IQR) | 11 (8-14) | 12 (9-14.75) | 11 (8-13) | <0.01 | 0.30 |
| APACHEII, median (IQR) | 27 (23-32) | 28 (23-33) | 27 (22-32) | 0.02 | 0.16 |
| Charlson, median (IQR) | 5 (3-7) | 6 (4-8) | 5 (3-7) | 0.01 | 0.18 |
| Ventilation^c^, n (%) | 779 (80.39) | 277 (78.25) | 502 (81.63) | 0.23 | 0.08 |
| Vasopressors^c^, n (%) | 344 (35.50) | 145 (40.96) | 199 (32.36) | 0.01 | 0.18 |

*Abbreviations: MAP: Mean arterial pressure; WBC: White blood cell; PT: Prothrombin Time; CKD: Chronic kidney disease; COPD: Chronic Obstructive Pulmonary Disease; SOFA: Sequential Organ Failure Assessment score; APACHEII: Acute Physiology and Chronic Health Evaluation II score; CCI: Charlson Comorbidity Index.*

*^a^ Total urine output in the 72 hours prior to CRRT.*

*^b^ The most recent fluid intake and output record and fluid balance record before CRRT.*

*^c^ Requirement of vasoactive drugs or mechanical ventilation on the first day of ICU admission.*

#GUID 272cbb65-9a09-47d3-a931-3aa214800222
