# Supplementary material for: Pre-CRRT furosemide and mortality in sepsis-associated AKI: A retrospective cohort study
Source: PLoS One. 2026 Apr 20;21(4):e0347094. doi: 10.1371/journal.pone.0347094 (PMC13095019; doi:10.1371/journal.pone.0347094)
Supplement: S6 Table — Abbreviations: MAP: Mean arterial pressure; WBC: White blood cell; PT: Prothrombin Time; CKD: Chronic kidney disease; COPD: Chronic Obstructive Pulmonary Disease; SOFA: Sequential Organ Failure Assessment score; APACHEII: Acute Physiology and Chronic Health Evaluation II score; Charlson: Charlson Comorbidity Index; HR: Heart rate; RR: Respiratory rate. (DOCX) [file pone.0347094.s015.docx]

**Table S6. The Univariable Cox regression results of 28-day all-cause mortality for the use of furosemide within 72 hours prior to CRRT in the unmatched cohort.**

| Variables | HR (95% CI) | *P*-value |
| --- | --- | --- |
| Age | 1.008 (1.001-1.016) | 0.022 |
| Albumin | 1.015 (0.879-1.171) | 0.843 |
| APACHEII | 1.019 (1.007-1.031) | <0.01 |
| Body temperature | 0.9 (0.748-1.082) | 0.261 |
| Calcium | 1.101 (1.006-1.204) | 0.037 |
| Cancer | 1.213 (0.883-1.667) | 0.234 |
| Charlson | 1.008 (0.977-1.039) | 0.622 |
| CKD | 0.895 (0.723-1.109) | 0.311 |
| COPD | 1.153 (0.898-1.482) | 0.265 |
| Creatinine | 0.916 (0.874-0.961) | <0.01 |
| Diabetes | 0.801 (0.662-0.969) | 0.022 |
| Ethnicity-White | 0.882 (0.655-1.187) | 0.407 |
| Ethnicity-Other | 1.377 (1.137-1.668) | <0.01 |
| Furosemide | 0.659 (0.550-0.790) | <0.01 |
| Male | 1.029 (0.856-1.237) | 0.763 |
| Heart faliure | 0.964 (0.794-1.170） | 0.712 |
| Height | 0.993 (0.984-1.003) | 0.159 |
| HR | 1.002 (0.998-1.007) | 0.264 |
| Hypertension | 1.012 (0.826-1.238) | 0.911 |
| Lactate | 1.020 (0.977-1.065) | 0.377 |
| MAP | 0.997 (0.991-1.002) | 0.209 |
| PH | 0.569 (0.274-1.183) | 0.131 |
| Phosphate | 1.016 (0.974-1.060) | 0.451 |
| Platelet | 0.998 (0.997-0.999) | <0.01 |
| Potassium | 1.050 (0.946-1.166) | 0.358 |
| PT | 1.042 (1.028-1.057) | <0.01 |
| RR | 1.011 (0.997-1.026) | 0.137 |
| Sodium | 0.994 (0.978-1.009) | 0.425 |
| SOFA | 1.040 (1.017-1.064) | <0.01 |
| Spo2 | 0.978 (0.955-1.003) | 0.08 |
| Urea_nitrogen | 0.998 (0.995-1.001) | 0.246 |
| Vasopressor | 1.143 (0.951-1.375) | 0.155 |
| Ventilation | 1.196 (0.947-1.510) | 0.133 |
| WBC | 1.014 (1.001-1.026) | 0.028 |
| Weight | 0.998 (0.994-1.002) | 0.235 |

*Abbreviations: MAP: Mean arterial pressure; WBC: White blood cell; PT: Prothrombin Time; CKD: Chronic kidney disease; COPD: Chronic Obstructive Pulmonary Disease; SOFA: Sequential Organ Failure Assessment score； APACHEII : Acute Physiology and Chronic Health Evaluation II score; Charlson: Charlson Comorbidity Index; HR: Heart rate; RR: Respiratory rate*
