# Supplementary material for: Pre-CRRT furosemide and mortality in sepsis-associated AKI: A retrospective cohort study
Source: PLoS One. 2026 Apr 20;21(4):e0347094. doi: 10.1371/journal.pone.0347094 (PMC13095019; doi:10.1371/journal.pone.0347094)
Supplement: S7 Table — Abbreviations: WBC: White blood cell; PT: Prothrombin Time; SOFA: Sequential Organ Failure Assessment score; APACHEII: Acute Physiology and Chronic Health Evaluation II score. (DOCX) [file pone.0347094.s016.docx]

**Table S7. The multivariate Cox regression results of 28-day all-cause mortality for the use of furosemide within 72 hours prior to CRRT in the unmatched cohort.**

| Variables | HR (95% CI) | *P*-value |
| --- | --- | --- |
| Age | 1.016 (1.008-1.025) | <0.01 |
| APACHEII | 1.015 (0.998-1.032) | 0.075 |
| Calcium | 1.077 (0.979-1.184) | 0.128 |
| Creatinine | 0.877 (0.831-0.925) | <0.01 |
| Platelet | 0.999 (0.998-1.000) | 0.043 |
| PT | 1.031 (1.015-1.047) | <0.01 |
| SOFA | 1.000 (0.967-1.033) | 0.986 |
| WBC | 1.016 (1.004-1.030) | 0.012 |
| Diabetes | 0.751 (0.612-0.922) | <0.01 |
| Ethnicity | 1.475 (1.213-1.795) | <0.01 |
| Furosemide | 0.591 (0.488-0.716) | <0.01 |

*Abbreviations: WBC: White blood cell; PT: Prothrombin Time; SOFA: Sequential Organ Failure Assessment score; APACHEII : Acute Physiology and Chronic Health Evaluation II score.*
