# Supplementary material for: Pre-CRRT furosemide and mortality in sepsis-associated AKI: A retrospective cohort study
Source: PLoS One. 2026 Apr 20;21(4):e0347094. doi: 10.1371/journal.pone.0347094 (PMC13095019; doi:10.1371/journal.pone.0347094)
Supplement: Attachment — Submitted filename: Response to Reviewers.docx [file pone.0347094.s018.docx]

**Response Letter**

**Manuscript Number:** PONE-D-25-46945

**Title:** *Pre-CRRT Furosemide and Mortality in Sepsis-Associated AKI: A Retrospective Cohort Study with Propensity Score Matching*

[Feb 28, 2026]

Dear Editor:

Thank you for giving us the opportunity to submit a revised draft of the manuscript “**Pre-CRRT Furosemide and Mortality in Sepsis-Associated AKI: A Retrospective Cohort Study with Propensity Score Matching (Manuscript Number: PONE-D-25-46945)**” for publication in *PLOS ONE*. We appreciate the time and effort that you and the reviewers dedicated to providing feedback on our manuscript and grateful for insightful comments and valuable improvements to our paper. We have incorporated these comments made by reviews. Other changes are highlighted in the manuscript. Please see below, in blue, for a point-by-point response to the reviewers’ comments and concerns. All page numbers refer to the revised manuscript file with tracked changes.

We hope that the changes we’ve made resolve all your concerns about the article. I’m more than happy to make any further changes that will improve the paper and facilitate successful publication.

Thank you and best regards.

Sincerely,

Zhong-Hua Lu

The First Department of Critical Care Medicine

The Second Affiliated Hospital of Anhui Medical University

678 Furong Road, Hefei, Anhui Province 230601, China

Email: luzhonghua077@126.com

**Journal Requirements:**

Dear Editor,

Thank you for your detailed instructions regarding the funding information. We have carefully addressed all points and provided the necessary clarifications below.

Regarding Requirements 3, 4, and 5 – Funding Information:

**Removal of funding text from the manuscript:**

We have removed all funding-related text from the Acknowledgments section and any other areas of the manuscript as requested. The Acknowledgments section now contains only non-funding acknowledgments.

**Updated Funding Statement:**

We request that the Funding Statement in the online submission form be updated to the following:

"This study was supported by the **Anhui Provincial Health and Health Research Project (Grant No. 2024Aa20292), the Anhui Province Traditional Chinese Medicine Inheritance and Innovation Research Project (Grant No. 2024CCCX128), the Special Research Fund for Analgesia and Sedation in Critical Care (Grant No. AHEBM20250707M1), and the Natural Science Research Project of Anhui Higher Education Institutions (Grant No. 2023AH053168)**."

**Role of funders:**

The funders had no role in study design, data collection and analysis, decision to publish, or preparation of the manuscript.

**Author salaries:**

No authors received salaries from any of the funders listed above.

**Consistency confirmation:**

We confirm that the funding information provided above is complete and accurate, and we request that this information be used to update both the "Funding Information" and "Financial Disclosure" sections in the online submission system to ensure consistency.

Please feel free to contact us if any further information is needed.

Sincerely,

Zhong-Hua Lu

**Comments from the Editor:**

Thank you for submitting your manuscript to PLOS ONE. After careful consideration, we feel that it has merit but does not fully meet PLOS ONE’s publication criteria as it currently stands. Therefore, we invite you to submit a revised version of the manuscript that addresses the points raised during the review process.

**Response:** We thank all the editors and reviewers for their valuable comments and suggestions. We have carefully revised the manuscript to enhance its clarity and facilitate the understanding of the readers. Our point-to-point responses are presented in the following. We hope that the revision would satisfactorily address the comments and concerns of the editors and reviewers.

**Reviewer #1**

This is a well-designed retrospective cohort study addressing an important clinical dilemma in the management of sepsis-associated AKI. The large sample size, consistent mortality findings across multiple analyses, and dose-response exploration are notable strengths. The manuscript contributes valuable real-world evidence to an area with limited data.

**Response: Thanks for the positive comments.**

**Comment 1: Causality and Interpretation**

The conclusions should be tempered to emphasize association rather than benefit. Statements implying safety or effectiveness should be clearly framed within the limitations of observational data.

**Response:** We sincerely appreciate this important criticism from the reviewer. The results of observational studies should be interpreted with caution as “association” rather than “causation”. To this end, we have systematically revised the relevant expressions throughout the manuscript. (See lines 69-75 in the abstract, 432-434in the discussion, and 450-458 in the conclusion of the revised draft for details)

lines 69-75 in the abstract: **These findings suggest that, in this selected cohort, pre-CRRT furosemide use was associated with lower mortality and does not appear to be associated with harm from delayed CRRT initiation. However, due to the observational design, these results should be warrant confirmation in prospective, randomized controlled trials.**

lines 432-434 in the discussion: Finally, **our findings demonstrate an association, not causation, and should not be interpreted as evidence of a direct beneficial effect of furosemide. The** detailed mechanisms underlying the observed association require further basic and clinical investigation.

lines 450-458 in the conclusion: In conclusion, this retrospective study suggests that pre-CRRT furosemide use **was associated with lower mortality in critically ill patients with SAKI. The findings provide reassurance that furosemide use prior to CRRT, even if it delays initiation, may not be associated with worse outcomes, and could potentially identify a subgroup of patients with a more favorable prognosis.** The benefits appear more pronounced in patients without pre-existing CKD and with higher furosemide doses. **Given the inherent limitations of the observational design,** these findings warrant validation in prospective, randomized controlled trials to establish causality and guide clinical practice.

**Comment 2:Residual Confounding**

Although propensity score matching was performed, residual differences in urine output and fluid balance remain clinically meaningful. These variables may reflect both treatment response and baseline severity and should be discussed more explicitly as potential mediators or confounders.

**Response:** Thank you for pointing out this crucial point. We have added a dedicated discussion in the “Limitations” paragraph of the discussion section. We acknowledge that urine output and fluid balance are not only indicators of treatment response but may also reflect unmeasured baseline disease severity. Despite the use of PSM, these variables may still serve as residual confounding factors or mediating factors, partially explaining the observed associations. This supplement aims to guide readers in interpreting the results more comprehensively and cautiously. (See lines 415-430 in the discussion of the revised draft for details)

lines 415-430 in the discussion: First, despite propensity score matching and multivariate adjustment, residual confounding may persist due to the observational nature of this study. **Notably, even after matching, clinically meaningful differences in urine output and fluid balance were observed between the furosemide and non-furosemide groups. These variables present a methodological challenge: they serve both as mediators of furosemide's therapeutic effect and as markers of underlying renal reserve and illness severity. Adjusting for them in outcome models could introduce overadjustment bias by 'conditioning on an intermediate,' potentially obscuring the true total effect of furosemide. Conversely, not accounting for them leaves open the possibility that the observed mortality association is partly explained by these residual differences, which may reflect unmeasured factors such as intrinsic renal responsiveness, adequacy of resuscitation, or subtle differences in tubular injury. Therefore, while our primary analysis employed PSM and multivariable Cox regression on a priori confounders, we cannot exclude the possibility that some residual confounding—particularly related to these physiologically intertwined variables—influences our findings.**

**Comment 3: Data Availability Compliance**

Please revise the Data Availability Statement to comply with PLOS ONE policy by explicitly stating that the data are derived from the MIMIC-IV database and providing access details.

**Response:** Thank you for this valuable suggestion. We have strictly adhered to the PLOS ONE data policy and rewritten the data availability statement. The original statement has been replaced with a more specific and compliant version. (See lines 489-498 in the data availability statement of the revised draft for details).

lines 489-498 in the data availability statement: **The data supporting this study are derived from the MIMIC-IV database (version 2.2), a publicly available resource hosted on PhysioNet (https://physionet.org/content/mimiciv/2.2/). Access to MIMIC-IV requires completion of a training course and signing of a data use agreement. The authors obtained access under credential ID 55377862. Per the data use agreement, the authors cannot redistribute the raw data; however, interested researchers may apply for access directly through PhysioNet at https://physionet.org/content/mimiciv/. All relevant analytic methods and study materials are described in the manuscript and its Supporting Information files.**

**Comment 4: CRRT Timing**

Since one of the main concerns addressed by the study is delayed CRRT initiation, providing more granular data or discussion on time-to-CRRT would further strengthen the manuscript.

**Response:** We sincerely appreciate the valuable comments provided by the reviewers. In response to their requests, we have included additional intergroup comparison data concerning the time interval from the diagnosis of SAKI to the initiation of CRRT. Our analysis indicates that the median time in the furosemide group was significantly longer than that in the non-furosemide group, thereby confirming the clinical observation of delayed CRRT initiation attributable to the use of diuretic therapy. These findings have been incorporated into a newly established subsection titled "Timing of CRRT Initiation" within the Results section, and we have thoroughly discussed the implications of this finding in the Discussion section. (See lines 65-66 in the Abstract, lines 304-311 in the results and lines 314-327 in the discussion of the revised draft for details)

lines 65-66 in the Abstract: **The use of furosemide is related to the prolongation of CRRT startup time (1.52 vs 1.46 days).**

lines 304-311 in the results:

**3.7. Timing of CRRT Initiation**

**Concerning the timing of CRRT initiation, the median duration from the diagnosis of SAKI to the commencement of CRRT was significantly longer in the furosemide group compared to the non-furosemide group, with values of 1.52 days (IQR 0.88-3.15) versus 1.46 days (IQR 0.58-1.89); p < 0.01. This observed difference equates to an approximate delay of 1.44 hours in CRRT initiation associated with the use of furosemide prior to CRRT (Table 3).**

Table 3 Differential analysis of time from SAKI diagnosis to initiation of CRRT.

| Group | | | | |
| --- | --- | --- | --- | --- |
| Variable | Overall | Non-Furosemide | Furosemide | *p*-value |
| N | 560 | 280 | 280 |  |
| Time of AKI to CRRT, (days), median (IQR) | 1.46 (0.75, 2.62) | 1.46 (0.58, 1.89) | 1.52 (0.88, 3.15) | <0.01 |

*IQR: Interquartile range*

lines 314-327 in the discussion: **In this cohort of SAKI patients requiring CRRT, pre-CRRT furosemide use was associated with significantly lower 28-day mortality—a finding that remained consistent across subgroup and sensitivity analyses. Despite the observed delay in CRRT initiation, this delay was not associated with increased mortality. This finding is reassuring, as it suggests that the practice of delaying CRRT to attempt diuresis does not appear to translate into net harm—at least within the 72-hour window defined in this study. This observation may be explained by the possibility that patients selected for furosemide represent a subgroup with greater renal reserve, and the diuretic trial itself may help identify those with a better underlying prognosis.**

**Comment 5: Minor Points:**

1. Improve language clarity and reduce repetition, especially in the Discussion.

2. Ensure consistent terminology (e.g., “pre-CRRT furosemide use” vs “furosemide administration within 72 hours”).

3. Consider adding a brief clinical implication section to guide readers on how findings may inform practice without overstating conclusions.

**Response:** We are grateful for the detailed suggestions provided by the reviewers. We have made modifications in three aspects: (1) We have conducted comprehensive text polishing for the entire article, especially the “Discussion” section, removing redundant and repetitive sentences to make the discussion smoother and more concise; (2) We have unified terminology throughout the article, consistently using “pre-CRRT furosemide use” as the core expression; (3) We have added a new “Clinical Significance” paragraph at the end of the Discussion section to provide practical guidance to readers without exaggerating the conclusions.(1&2 see the revised draft for details;3 see lines 435-448 in the discussion of the revised draft for details)

lines 435-448 in the discussion: **While causality cannot be inferred from this observational study, the findings offer several provisional insights for clinicians. First, in patients with SAKI being considered for CRRT, a trial of furosemide—when not contraindicated—may be reasonable, as it was not associated with harm even among those who ultimately required RRT. Second, the more pronounced benefit observed in patients without pre-existing CKD suggests that furosemide responsiveness may help identify individuals with greater renal reserve who could potentially avoid or delay CRRT. Third, higher furosemide doses (>90 mg/72h) were associated with greater mortality reduction, although this must be balanced against the known dose-dependent risks of electrolyte disturbances and ototoxicity. Importantly, these findings should not be interpreted as supporting routine high-dose furosemide, but rather as hypothesis-generating observations that require confirmation in randomized trials. Pending such evidence, clinical judgment regarding volume status, hemodynamic stability, and individual patient characteristics should guide pre-CRRT diuretic use.**

**Reviewer #2:**

This is a very good and novel study that addresses an important clinical question. The study is well designed, methodologically sound, and provides valuable insights with potential implications for clinical practice.

**Response: Thanks for the positive comments.**

**Comment 1： Score Matching**

Line 2-3: Very interesting title, but you don’t need to mention the statistical tests used in the study (Propensity Score Matching).

**Response:** We appreciate the reviewer's insightful suggestion. We agree that the title should focus on the core scientific issue of the study, rather than the specific statistical method used. Propensity Score Matching (PSM) is an analytical method adopted in this study, and mentioning it in the title would appear redundant. Therefore, we will remove “with Propensity Score Matching” from the title to make it more concise and focused.(see lines 2-5 in the title of the revised draft for details)

lines 2-5 in the title: Pre-CRRT Furosemide and Mortality in Sepsis-Associated AKI: A Retrospective Cohort Study

**Comment 2：**

Line 24-29:Specify the precise objectives of your study; currently, you only state the aim.

**Response:** Thank you for the reviewer's suggestions. We agree that the research objective in the abstract needs to be more specific and clear. We have refined the original general expression into specific research goals, clearly specifying the primary endpoint, secondary endpoint, and exploratory analysis. (see lines 33-41 in the Objective of the revised draft for details)

lines 33-41 in the Objective : **We aimed to assess whether pre-CRRT furosemide use in SAKI patients requiring CRRT is associated with 28-day mortality, and to evaluate its relationship with secondary outcomes including short-term mortality, in-hospital death, and length of stay. We also examined potential effect modification by CKD status and furosemide dosage.**

**Comment 3：**

Line 76:The introduction is very well written, clear, and effectively aligned with the study's core objectives. I suggest adding a paragraph that discusses the potential mechanisms by which pre-CRRT furosemide administration might influence mortality, to strengthen the clinical rationale and contextualize the study findings.

**Response:** Thank you for the reviewer's affirmation and suggestions. We agree and have added a discussion on the potential mechanism by which furosemide may affect prognosis in the introduction section. (see lines 93-96 in the Introduction of the revised draft for details)

lines 93-96 in the Introduction: **The rationale for this practice is supported by several potential mechanisms. By reducing fluid overload，furosemide may alleviate pulmonary edema, improve oxygenation, and facilitate liberation from mechanical ventilation^[13]^. Conversely, furosemide use carries inherent risks, including electrolyte disturbances and ototoxicity^[14]^.**

**Comment 4:**

Line 82:I recommend adding a dedicated subsection titled *“Study design, participants, and setting.” Additionally, the study design is not clearly described and should be explicitly stated to improve clarity and methodological transparency.

Line 94:

Include this criterion within the inclusion criteria (Line 87).

**Response:** Thank you for your invaluable suggestions. We have emphasized the original subsection heading “2.1. Study design, participants, and setting”. The study design has been rephrased for enhanced clarity and precision. Additionally, we have relocated the statement “For patients with multiple ICU hospitalizations, only the first admission episode was analyzed” to the inclusion criteria section, thereby presenting it as a definitive inclusion condition to ensure logical integrity. (see lines 113-140 in the Methods of the revised draft for details)

lines 113-140 in the Methods: **This was a retrospective cohort study using data from the Medical Information Mart for Intensive Care (MIMIC-IV) database, version 2.2. MIMIC-IV contains de-identified electronic health records from 534,912 critical care admissions at Beth Israel Deaconess Medical Center (Boston, MA, USA) between 2008 and 2019.**

The demand for CRRT primarily arises in critically ill patients suffering from severe AKI. In light of this clinical context and the inherent limitations in patient distribution within the database, **We included critically ill adult patients who (1) met the diagnostic criteria for sepsis and stage 3 AKI; (2) received CRRT during their ICU stay; and (3) were admitted to the ICU for the first time during the study period (for patients with multiple ICU admissions, only the first hospitalization was analyzed). Sepsis was defined according to the Third International Consensus Definitions (Sepsis-3), requiring suspected or confirmed infection accompanied by an acute increase of ≥2 points in the Sequential Organ Failure Assessment (SOFA) score. AKI was staged based on the KDIGO 2012 criteria: an increase in serum creatinine (SCr) of ≥0.3 mg/dL (26.5 μmol/L) within 48 hours, a ≥1.5-fold increase from baseline within 7 days, or urine output <0.5 mL/kg/h for ≥6 hours^[17]^.**

The exclusion criteria are as follows : **(1) age <18 or >75 years; (2) ICU stay <48 hours;** and (3) other diuretics besides furosemide were used.

**Comment 5:**

Line 136: For Figure 1, the propensity score matching flow diagram is conceptually appropriate and consistent with a 1:1 matching approach. However, the figure lacks information on excluded unmatched patients and should be complemented by a clear description of the matching methodology and balance diagnostics to ensure transparency and reproducibility.

**Response:** We fully endorse your suggestion. We have redrawn Figure 1 (the patient screening flowchart). The new flowchart clearly illustrates the complete process from initial screening, application of exclusion criteria, to propensity score matching. We have also provided more detailed descriptions in both the figure legend and the Methods section regarding the specific PSM parameters (nearest-neighbor algorithm; 1:1 matching; caliper value=0.05) and balance diagnostic criteria (SMD<0.10). (see Fig 1 and lines 175-177 in the methods of the revised draft for details)

lines 175-177 in the methods: Propensity score matching (**nearest-neighbor algorithm** ; 1:1 **matching**; caliper=0.05) was performed using variables unrelated to urine output or fluid balance, achieving balance (SMD<0.10)**^[23]^**.

Fig 1

Fig. 1 Figure 1 Flow chart of patient selection. *AKI: Acute kidney injury; CRRT: continuous renal replacement therapy ICU: intensive care unit; MIMIC-IV: Medical Information Mart for Intensive Care IV; SAKI: Sepsis-associated Acute kidney injury; PSM: propensity score matching.*

**Comment 6:**

Line 140: Please revise the manuscript to report baseline characteristics for the propensity score–matched cohort and present them after propensity score matching, as this approach is essential for reducing bias and controlling for confounding. Baseline characteristics before matching may also be reported; however, the primary focus should be on the post-matching results.

**Response:** Thank you for highlighting this crucial point. We have reorganized the “Baseline Characteristics” section: first briefly describing the significant differences present before matching, then focusing the description entirely on the characteristics of the matched cohort. (see lines 199-225 in the “Baseline characteristics” of the revised draft for details)

lines 199-225 in the Baseline characteristics: **Baseline characteristics of the study population before and after propensity score matching are presented in Supplementary digital content 6 and Table 1. Before matching, significant between-group differences existed in multiple variables, including demographics, vital signs, laboratory values, severity scores, and comorbidities, reflecting confounding by indication (Supplementary digital content 6). After 1:1 propensity score matching, 280 patients with pre-CRRT furosemide use were successfully matched to 280 patients without furosemide use. The matching procedure achieved excellent covariate balance, with absolute standardized mean differences (SMD) below 0.10 for all variables except serum creatinine, which showed a minimal residual imbalance (SMD = 0.10) (Table 1; Supplementary digital content 7). In the matched cohort, the two groups were well-balanced across all measured baseline characteristics, including age, gender, severity scores (SOFA, APACHE II), comorbidities, and key laboratory parameters. The distribution of propensity scores before and after matching is illustrated in Supplementary digital content 8.**

**Comment 7:**

Line 176: 3.5. Subgroup analyses:

Please report the results directly with their statistical significance. Avoid interpreting confidence intervals or p-values. The Results section should focus on presenting the findings rather than discussing their statistical implications.

**Response:** Thank you for your valuable suggestions. We have revised the presentation in Section 3.5 “Subgroup Analysis” to directly report the hazard ratios (HRs) with 95% confidence intervals for each subgroup, along with the *p*-values for interaction. All interpretive language has been removed, retaining only factual reporting of HR values, confidence intervals, *p*-values, and interaction *p*-values. (see lines 276-286 in the “Subgroup analyses” of the revised draft for details)

lines 276-286 in the “Subgroup analyses”: **Figure 3** displays the results of subgroup analysis for 28-day all-cause mortality in the matched cohort. **In patients without pre-existing CKD,** **pre-CRRT furosemide use** was associated with a lower mortality (HR, 0.53; 95% CI, 0.43-0.66; *p*<0.001). The *p*-value for interaction between furosemide use and CKD status **was 0.021.** **For all other subgroups examined—including age, sex, SOFA score, APACHE II score, and other comorbidities—the HR point estimates for furosemide use** were also below 1.00, **with** the p-values for interaction **exceeding** 0.05.

**Comment 8:**

Line 185: 3.6. Sensitivity analyses:

I believe there is redundancy between this section and lines 154–158, which makes the presentation confusing. Please clarify how this section differs from lines 154–158 or consider consolidating the overlapping content to improve clarity, or combine with primary outcome paragraph.

**Response:** We fully endorse your perspective. To eliminate confusion, we have removed the original standalone "Sensitivity Analysis" subsection. In the primary outcomes section, we first report the main findings from the matched cohort, followed by an explanation that sensitivity analyses were conducted in the unmatched cohort with consistent results. (see lines 237-250 in the “Primary outcome” of the revised draft for details)

lines 237-250 in the “Primary outcome”: The 28-day all-cause mortality rate in the furosemide group was 44.29% (124/280), compared to 58.57% (164/280) in the non-furosemide group. Figure 2 presents the Kaplan-Meier curves for 28-day all-cause mortality based on **pre-CRRT** furosemide use in the matched cohort. **Both univariate and multivariable Cox regression confirmed a significant association between pre-CRRT furosemide use and reduced 28-day mortality (univariate HR 0.60, 95% CI 0.48-0.76; multivariable HR 0.58, 95% CI 0.46-0.73; both p<0.01).** **To assess the robustness of this finding, we performed sensitivity analyses in the full unmatched cohort (N=969). These analyses yielded consistent results: furosemide use was associated with lower 28-day mortality in both univariate (HR, 0.66; 95% CI, 0.55-0.79; p<0.001) and multivariable models (HR, 0.59; 95% CI, 0.49-0.72; p<0.001) (Supplementary digital content 5). The corresponding Kaplan-Meier curve is shown in Supplementary digital content 9.**

**Comment 9:**

Line 192: 3.7. Dosage and Prognosis:

Please clarify the furosemide dosing strategy, specifically whether it was administered as a continuous infusion over 24 hours or as intermittent dosing. This distinction is important, as it remains an area of debate in the literature and may influence clinical outcomes.

**Response:** Thank you for your important feedback. The administration mode of furosemide (continuous infusion vs. intermittent push injection) is a key factor affecting pharmacodynamics and clinical outcomes, and is also a focus of ongoing controversy in the literature. However, although the MIMIC-IV database records administration events, it does not clearly distinguish between administration methods (continuous infusion vs. intermittent push injection) and infusion duration. Therefore, we are unable to conduct a precise analysis of this variable in this study. We will delve into the potential impact of drug administration methods in the discussion section, as well as how future research should be designed to address this issue. (see lines 405-413 in the discussion of the revised draft for details)

lines 405-413 in the discussion : **However, existing evidence suggests that the clinical benefits of furosemide are balanced against its potential risks in a dose-dependent manner. High-dose application may significantly increase the risk of adverse events such as electrolyte disturbances, hypovolemia, and worsening renal function^[43]^. The observed dose-response relationship in our study may therefore reflect a composite of different administration strategies, and we cannot exclude the possibility that the association between dose and outcome differs by infusion modality. Future studies with granular data on administration route and duration are needed to clarify whether the method of furosemide delivery modifies its effect on mortality in SAKI patients.**

**Comment 10:**

Line 213 – 216:Please indicate whether there are previous studies worldwide that have assessed these outcomes (7-day, 90-day, ICU, and in-hospital mortality) in relation to furosemide use, and briefly contextualize the findings in relation to your findings.

**Response:** We appreciate your valuable suggestions. In response, we have incorporated a new section in the Discussion that examines whether other studies worldwide have assessed the relationship between furosemide and mortality rates at 7 days, 90 days, in the ICU, and during hospitalization. Additionally, we briefly compare these findings with the results of our study. It is noteworthy that some prior research has also indicated an association between furosemide and reduced mortality. However, studies focusing on 7-day mortality and ICU mortality are still relatively scarce, underscoring the complementary value of our research. (see lines 347-355 in the discussion of the revised draft for details)

lines 347-355 in the discussion: **These findings not only corroborate but also expand upon previous observations. Zhao et al. reported in a large cohort of AKI patients from the MIMIC-III database that the use of furosemide was associated with a reduction in both in-hospital and 90-day mortality rates^[12]^. Similarly, Li et al., in their study of SAKI patients undergoing RRT within the MIMIC-IV database, found that furosemide use correlated with decreased in-hospital mortality[24]. However, data regarding 7-day mortality—outcomes that may more accurately reflect the immediate effects of diuretic therapy and the timing of CRRT—remains limited. Our study provides novel evidence indicating that the use of furosemide prior to CRRT is linked to these early mortality outcomes, thereby reinforcing the potential advantages of this treatment strategy.**

We sincerely appreciate the valuable time and insightful comments provided by all the editors and reviewers, which have significantly enhanced the quality of our manuscript.
